# A novel metric reveals biotic resistance potential and informs predictions of invasion success

**DOI:** 10.1038/s41598-019-51705-9

**Published:** 2019-10-25

**Authors:** Ross N. Cuthbert, Amanda Callaghan, Jaimie T. A. Dick

**Affiliations:** 1 Institute for Global Food Security, School of Biological Sciences, Queen’s University Belfast, Belfast, BT9 5DL UK; 2 Ecology and Evolutionary Biology, School of Biological Sciences, University of Reading, Harborne Building, Reading, RG6 6AS UK

**Keywords:** Food webs, Invasive species

## Abstract

Invasive species continue to proliferate and detrimentally impact ecosystems on a global scale. Whilst impacts are well-documented for many invaders, we lack tools to predict biotic resistance and invasion success. Biotic resistance from communities may be a particularly important determinant of the success of invaders. The present study develops traditional ecological concepts to better understand and quantify biotic resistance. We quantified predation towards the highly invasive Asian tiger mosquito *Aedes albopictus* and a representative native mosquito *Culex pipiens* by three native and widespread cyclopoid copepods, using functional response and prey switching experiments. All copepods demonstrated higher magnitude type II functional responses towards the invasive prey over the analogous native prey, aligned with higher attack and maximum feeding rates. All predators exhibited significant, frequency-independent prey preferences for the invader. With these results, we developed a novel metric for biotic resistance which integrates predator numerical response proxies, revealing differential biotic resistance potential among predators. Our results are consistent with field patterns of biotic resistance and invasion success, illustrating the predictive capacity of our methods. We thus propose the further development of traditional ecological concepts, such as functional responses, numerical responses and prey switching, in the evaluation of biotic resistance and invasion success.

## Introduction

Invasive alien species continue to threaten biodiversity globally and disrupt ecosystem structure and functioning^1,2^. Identifying and understanding ecological processes that contribute to invasion success are essential to predicting and remediating invader impacts in communities^3,4^. In particular, species diversity in recipient communities can drive ‘biotic resistance’ towards invasive species as a result of competition, predation, parasitism and other antagonisms, thus potentially limiting or preventing invasion success^5,6^. Predation is an especially pervasive force which can shape ecosystem structuring through both trait- and density-mediated interactions with prey^7,8^. However, the relationship between the invasibility of communities and levels of biotic resistance (e.g. predation) therein remain unclear, and the development and validation of methodologies to predict invasion success or failure are urgently required^4^. Indeed, the identification of universal species traits which reliably predict invader impact and success across taxonomic and trophic groups has thus far been largely unsuccessful^9^. In turn, this has acted as a hindrance to practical assessments of invasion risk and the associated development of measures to remediate invader impacts, and particularly for invasive species with no known invasion history^3^.

Invasion science has been slow to integrate some key ecological concepts which are classically predictive in consumer-resource systems^3^. Traditionally, the functional response, defined as the relationship between resource availability and resource use, has been applied by ecologists to quantify interaction strengths between consumers and resources (e.g. predators and prey)^10–12^. Indeed, both functional response form and magnitude have been identified as robust measures of ecological impact from consumers towards resources, including from existing and emerging invasive species towards native resources^4,9,13,14^. In particular, the attack rate (search coefficient) and handling time parameters of functional response models align closely with the magnitude of ecological impacts across resource densities^9^. Furthermore, whilst three broad forms of functional response have generally been categorised (linear type I, saturating type II, sigmoidal type III), type II functional responses have been identified as particularly impactful towards target resources due to the destabilising pressures they impart on low densities of resources such as prey species^13^. In contrast, type III functional responses may enable population stability of resources due to low density refuge effects^15^.

Classically, the consumer functional response has also been combined with the numerical response, which describes the consumer population response as resource densities change. In turn, this can quantify the overall impact of consumers on resource populations, termed the total response or offtake rate^10–12^. Recently, the functional response approach in invasion science has been combined with proxies for the numerical response (e.g. abundance, fecundity) to develop population-level metrics which have been proven to correlate tightly with known ecological impacts (i.e. Relative Impact Potential^3^; Relative Invasion Risk^16^). However, whilst functional and numerical responses have been applied to examine the ecological impacts of invasive consumers (e.g. predators) towards native resources (e.g. prey), there has been relatively little application of these approaches to quantify biotic resistance potential towards invasive resources (e.g. prey) in the context of invasion success^6,17,18^.

Aside from functional and numerical responses, integrations of switching propensities between invasive and native resources have been neglected by invasion scientists^4,19–21^. This is despite the importance of consumer switching patterns for the persistence of species. Characteristically, if a consumer exhibits a switching propensity, disproportionately fewer rare resources are consumed whilst, simultaneously, disproportionately more abundant resources are consumed^15^. Importantly, switching (i.e. a form of frequency-dependent predation) may facilitate coexistence between resources (e.g. prey) by imparting stability through low-density refugia concomitant with type III functional responses^22^. Therefore, in the context of invasion science, switching propensities by resident consumers have the capacity to directly influence biotic resistance towards invasive species and could thus be used in combination with functional and numerical responses of consumers in predictive approaches for invasion success. Indeed, if a resident consumer exhibits high magnitude *per capita* (functional response) and population-level (numerical response) effects, coupled with consumptive preferences towards invasive resources, offtake rates towards invaders will be high and it is thus theoretically less likely that the invader will succeed and subsequently have impacts on ecosystems. Although mechanistic interpretation must be cautioned in the absence of field experiments, comparative laboratory experiments may be used to rapidly quantify these effects (i.e. functional/numerical responses and preferences), and such studies have proven highly informative in the context of invader impact and success^14,20^.

The present study develops a novel approach to quantify and compare levels of biotic resistance by resident consumers towards invasive species using the aforementioned concepts. We have three key objectives: (1) to quantify *per capita* effects among natural enemies towards separate invasive and native prey; (2) to examine ratio-dependent prey preferences of the natural enemies where invasive and native prey coexist, and; (3) to use population-level responses alongside *per capita* effects and prey preferences to quantify and compare levels of biotic resistance among agents. These results help to inform management responses for the biocontrol of target species by providing a novel means of comparing agent efficacies. Further, our results help to inform predictions of invasion success, given that high levels of biotic resistance towards invasive over native species may limit invasive species success likelihoods^5,6^.

We use a well-documented field pattern, whereby an invasive mosquito species is a superior competitor compared to natives within the same trophic level, but is known to coexist, perhaps through differential biotic resistance from indigenous predators^23–26^. We develop our approach using a model invader/native system based on the invasive Asian tiger mosquito *Aedes albopictus* and native common house mosquito *Culex pipiens*. *Aedes albopictus* is a highly invasive species that can vector pathogens that cause disease, and is known to outcompete native mosquitoes^27–29^. Yet, this invader has been documented to coexist with native species in aquatic habitats despite its superior competitive ability^24^. Whilst *C. pipiens* is also regarded as an invasive vector species in certain parts of the world^30^, in our study system (United Kingdom) it represents a widespread and abundant native species^31^, and is thus an appropriate candidate for invader-native comparative purposes. We quantify and compare biotic resistance towards these mosquito prey by three species of cyclopoid copepods. Predatory copepods are abundant and widespread crustaceans which are capable of thriving in most aquatic habitat types^32^. Given their tolerance to ephemeral environments *via* dormant life stages, and potential for both human-mediated and zoochorous dispersal between aquatic habitats^33–36^, there is high potential for copepod-mosquito overlap in various aquatic habitat types and thus predatory interactions. Furthermore, copepods are known to regulate mosquito populations in aquatic systems^36^. Informed by empirical field-patterns, we hypothesise that: (1) copepod functional response magnitudes will be significantly greater towards invasive compared to native mosquito prey; (2) predators will display a ratio-independent preference for invasive mosquito prey over native mosquitoes; (3) biotic resistance from native natural enemies will differ among predator species according to their *per capita*, selectivity and population-level effects.

## Results

**Figure 1:**
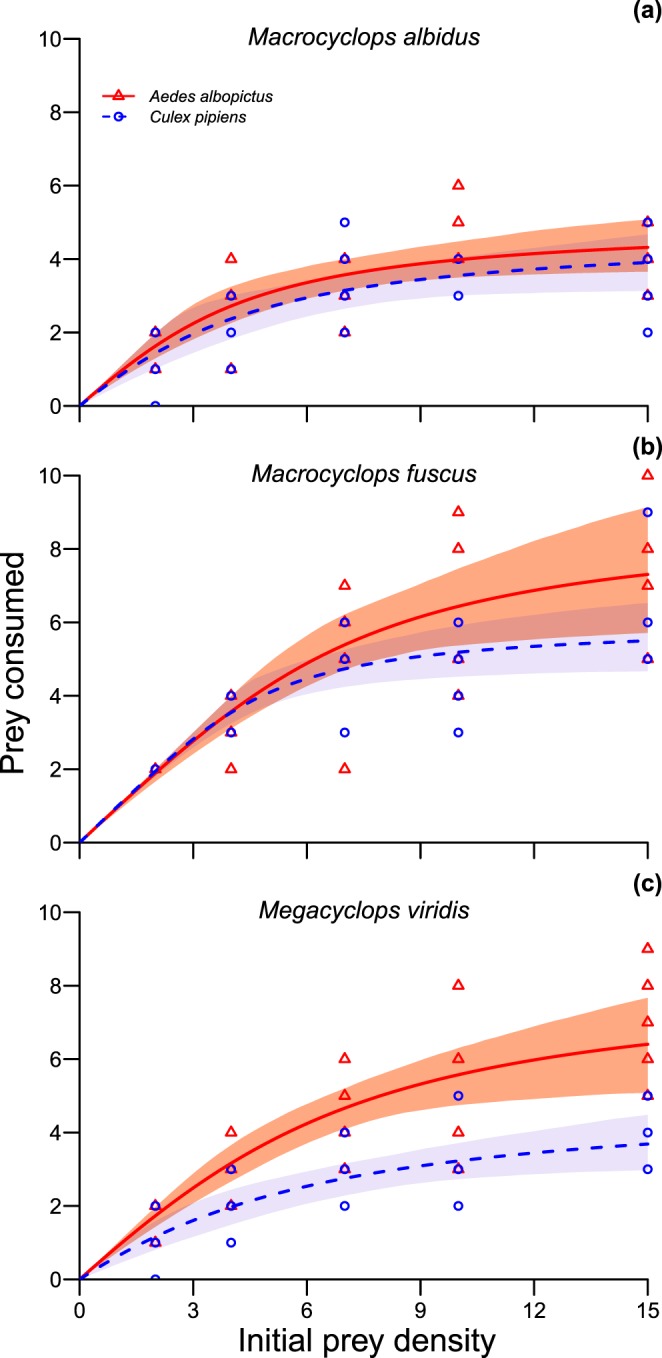
Functional responses of *Macrocyclops albidus* (**a**), *Macrocyclops fuscus* (**b**) and *Megacyclops viridis* (**c**) towards *Aedes albopictus* (red triangles) and *Culex pipiens* (blue circles) prey. Shaded areas are bootstrapped (*n* = 2000) 95% confidence intervals and points are underlying consumption data.

In all experiments, 100% of control prey survived and thus experimental deaths were directly attributable to predation by copepods, which was also evidenced by partially consumed prey remaining post-experiment. In the functional response experiment, support for raw consumption models containing prey species, predator species and prey density received substantial support (Table S1a). Significantly greater numbers of invasive *A. albopictus* were consumed as compared to native *C. pipiens* overall (χ^2^ = 9.14, df = 1, p = 0.003; Fig. 1). Consumption differed significantly across predator species (χ^2^ = 16.11, df = 2, p < 0.001), owing to significantly greater consumption by *M. fuscus* than *M. albidus* (p < 0.001) and *M. viridis* (p = 0.02). However, consumption towards *A. albopictus* was higher for all predators given a statistically unclear ‘prey species × predator species’ interaction (χ^2^ = 4.19, df = 2, p = 0.12). Consumption was also significantly greater under increasing prey densities (χ^2^ = 73.66, df = 4, p < 0.001).

All predatory copepods exhibited type II functional responses towards both *C. pipiens* and *A. albopictus*, owing to significantly negative first order linear coefficients (Fig. 1; Table 1). Attack rates tended to be higher towards *A. albopictus* than *C. pipiens* prey by *M. albidus* and *M. viridis*, whilst handling times were generally shorter for *A. albopictus* across all predator species (Table 1; Fig. 1). Maximum feeding rates were thus higher towards invasive *A. albopictus* prey than native *C. pipiens* for all three predators (Fig. 1; Table 1). Accordingly, in all instances, overall *per capita* predatory impacts as quantified by the functional response ratio (FRR) were considerably higher towards the invasive *A. albopictus* over the native *C. pipiens* (Table 1). Differential *per capita* predatory impacts between prey were, however, particularly pronounced for *M. viridis* (Table 1), and further evidenced by a divergence in confidence intervals under higher prey densities (Fig. 1c), as compared to confidence interval convergence in *M. albidus* (Fig. 1a) and *M*. *fuscus* treatments (Fig. 1b).

**Table 1:** Functional response linear coefficients, parameters and associated significance levels of *Macrocyclops albidus*, *Macrocyclops fuscus* and *Megacyclops viridis* towards larval *Aedes albopictus* and *Culex pipiens* prey.

Predator	Prey	Linear coefficient, p	Attack rate (*a*), p	Handling time (*h*), p	Maximum feeding rate (1/*h*)	Functional response ratio (FRR: *a*/*h*)
*Macrocyclops albidus*	*Aedes albopictus*	−0.160, <0.001***	2.507, 0.015*	0.200, <0.001***	5.000	12.535
*Macrocyclops albidus*	*Culex pipiens*	−0.134, <0.001***	1.820, 0.020*	0.214, <0.001***	4.673	8.505
*Macrocyclops fuscus*	*Aedes albopictus*	−0.183, <0.001***	3.726, 0.001**	0.112, <0.001***	8.929	33.268
*Macrocyclops fuscus*	*Culex pipiens*	−0.185, <0.001***	5.285, 0.017*	0.166, <0.001***	6.024	31.837
*Megacyclops viridis*	*Aedes albopictus*	−0.124, <0.001***	2.549, 0.003**	0.122 < 0.001***	8.197	20.893
*Megacyclops viridis*	*Culex pipiens*	−0.095, 0.009**	1.139, 0.022*	0.204, <0.002**	4.902	5.583

Asterisks denote significance levels (*<0.05, **<0.01, ***<0.001).

**Figure 2:**
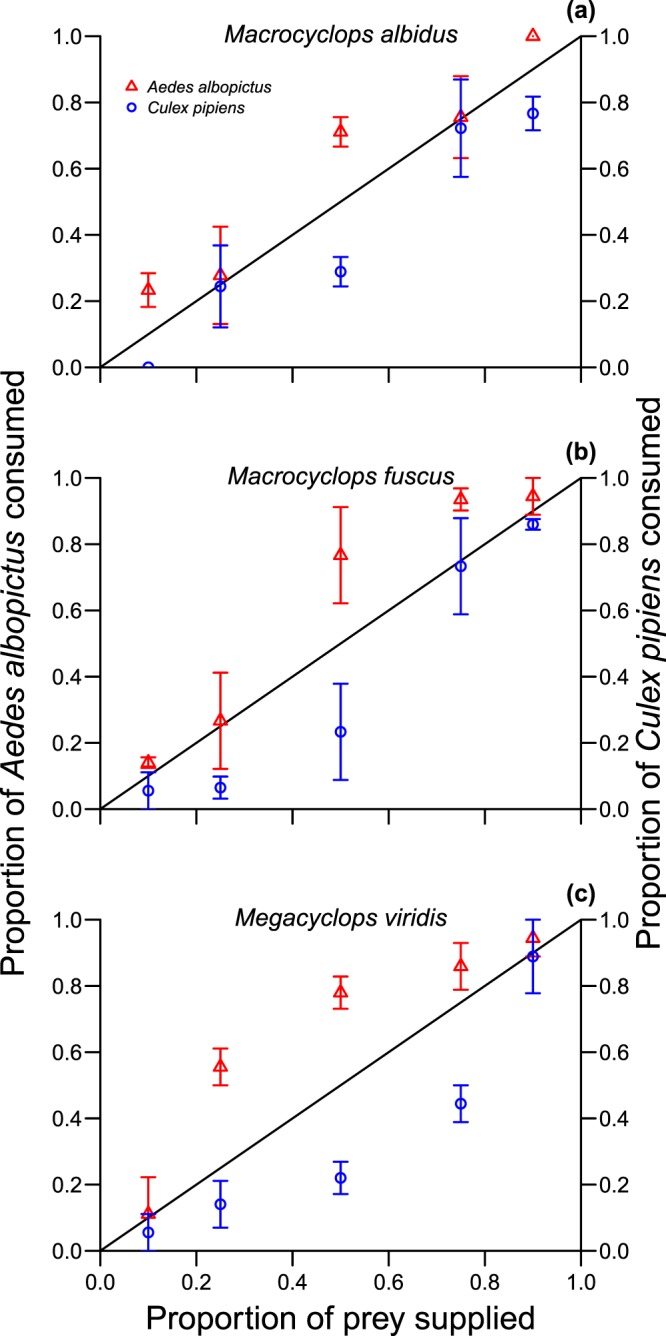
Prey switching propensities of *Macrocyclops albidus* (**a**), *Macrocyclops fuscus* (**b**) and *Megacyclops viridis* (**c**) towards different proportions of *Aedes albopictus* (red triangles) and *Culex pipiens* (blue circles) prey. The solid line indicates the expected value in the case of no preference between prey types. Means are ± 1 SE.

Raw consumption in the prey switching experiments was also substantially influenced by prey species, predator species and proportional prey availability (Table S1b). Again, significantly greater numbers of invasive *A. albopictus* were consumed than native *C. pipiens* (χ^2^ = 17.14, df = 1, p < 0.001; Fig. 2). Consumption was significantly influenced by predator species (χ^2^ = 7.48, df = 2, p = 0.02), owing to significantly greater consumption by *M. fuscus* as compared to *M. albidus* (p = 0.04), and was greater where a particular prey species was present in higher proportions (χ^2^ = 70.08, df = 4, p < 0.001).

None of the focal copepod predators exhibited a prey switching propensity between invasive *A. albopictus* and native *C. pipiens* (Table 2; Fig. 2). Instead, preferential selection towards the invader was exhibited across all proportional availabilities, as evidenced by recurrently high preference indices (*a*_*i*_ > 0.5; Table 2). Preference index type (predicted/observed) and proportional availability were identified as important model components (Table S1c). Preference indices towards *A. albopictus* were significantly higher than expected under conditions of no preference (χ^2^ = 16.33, df = 1, p < 0.001). However, the strength of preference towards *A. albopictus* interacted with its proportional availability (χ^2^ = 17.10, df = 4, p = 0.002). Here, overall, preferences towards *A. albopictus* were particularly stronger than expected under higher proportional availabilities (0.5, 0.75, 0.9) (all p ≤ 0.01).

**Table 2:** Manly’s selectivity indices towards different proportional availabilities of *Aedes albopictus* by *Macrocyclops albidus*, *Macrocyclops fuscus* and *Megacyclops viridis*.

Predator	Proportion *Aedes albopictus* available	Preferences (*a*_*i*_) (±SE)
*Macrocyclops albidus*	0.10	0.756 ( ± 0.052)
*Macrocyclops albidus*	0.25	0.475 ( ± 0.244)
*Macrocyclops albidus*	0.50	0.735 ( ± 0.047)
*Macrocyclops albidus*	0.75	0.565 ( ± 0.219)
*Macrocyclops albidus*	0.90	1.000 ( ± 0.000)
*Macrocyclops fuscus*	0.10	0.618 ( ± 0.041)
*Macrocyclops fuscus*	0.25	0.466 ( ± 0.241)
*Macrocyclops fuscus*	0.50	0.776 ( ± 0.147)
*Macrocyclops fuscus*	0.75	0.876 ( ± 0.066)
*Macrocyclops fuscus*	0.90	0.773 ( ± 0.227)
*Megacyclops viridis*	0.10	0.285 ( ± 0.285)
*Megacyclops viridis*	0.25	0.830 ( ± 0.026)
*Megacyclops viridis*	0.50	0.816 ( ± 0.052)
*Megacyclops viridis*	0.75	0.711 ( ± 0.145)
*Megacyclops viridis*	0.90	0.773 ( ± 0.227)

Values greater than 0.5 indicate selective preference for *A. albopictus* prey.

**Figure 3:**
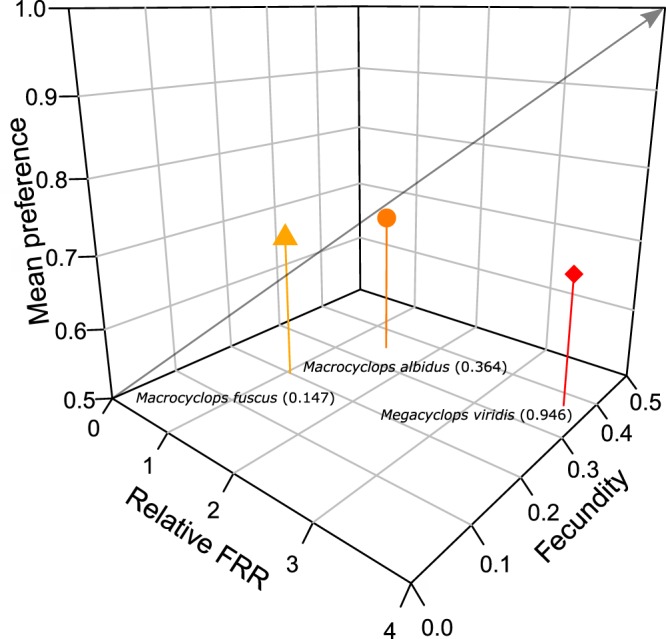
Triplot illustrating differential Biotic Resistance (BR) of *Macrocyclops albidus* (circle), *Macrocyclops fuscus* (triangle) and *Megacyclops viridis* (diamond) towards invasive *Aedes albopictus* prey. Estimations include relative functional response ratios (FRRs) between invasive and native prey, predator fecundities and invasive prey preferences. Increasing levels of BR are read from bottom left to top right, and raw BR scores are displayed in parentheses. Colours are ramped with increasing BR towards red colouration.

Owing to greater relative FRR between invasive/native prey species, higher fecundity and strong preferences towards invasive *A. albopictus* prey, Relative Biotic Resistance (RBR) was greater by *M. albidus* as compared to *M. fuscus* (Table 3). However, in turn, RBR scores of both *M. albidus* and *M. fuscus* were lower than *M. viridis*. This was driven by particularly marked relative FRR of *M. viridis* towards *A. albopictus* prey, coupled with higher levels of fecundity in this copepod (Table 3; Fig. 3). Accordingly, *M. viridis* is expected to exert the greatest degree of biotic resistance towards the invasive prey.

**Table 3:** Relative Biotic Resistance (RBR) levels between copepod groups towards invasive *Aedes albopictus*, derived from the multiplication of relative *per capita* impacts between *A*.

Comparators (predator 1, predator 2)	Relative FRR (FRR_*i*_/FRR_*n*_) (predator 1, predator 2)	Fecundity (FE) (predator 1, predator 2)	Mean preference (*a*_*i*_) (predator 1, predator 2)	Biotic Resistance (BR) (predator 1, predator 2)	Relative Biotic Resistance (RBR) (predator 1 vs. predator 2)
*Macrocyclops albidus, Macrocyclops fuscus*	1.474, 1.045	0.350, 0.200	0.706, 0.702	0.364, 0.147	2.476
*Macrocyclops albidus, Megacyclops viridis*	1.474, 3.742	0.350, 0.370	0.706, 0.683	0.364, 0.946	0.385
*Macrocyclops fuscus, Megacyclops viridis*	1.045, 3.742	0.200, 0.370	0.702, 0.683	0.147, 0.946	0.155

*albopictus* (FRR_*i*_) and native *Culex pipiens* (*FRR*_*n*_) prey, predator reproductive effort (fecundity; clutch weight produced per female body weight per day^47^) and mean preference indices towards *A. albopictus* across prey proportions available. Relative Biotic Resistance values of 1 indicate equivalent impacts between predators, whilst values >1 indicate greater relative impact, and values <1 lesser impact, of predator 1 as compared to predator 2.

## Discussion

Invasive alien species continue to spread, establish and reproduce in novel environments^1^, yet we have a distinct lack of methodologies to predict invasion success^3,4^. Biotic resistance may be a key mechanism which controls the success of invasive species. In the present study, we develop novel measures of biotic resistance driven by resident consumers towards invasive prey through the integration of functional responses, numerical response proxies and prey switching propensities. Our model species *Aedes albopictus* is a highly invasive vector mosquito, known to have superior competitive abilities for resources over analogous native mosquitoes, however often fails to displace these natives^24,27,29,37^. *Culex pipiens* is a native and widespread mosquito in our study region (United Kingdom), whilst *A. albopictus* has only recently been detected in the United Kingdom, following numerous successful invasions across Europe^38^. However, our results suggest that differential biotic resistance may limit the invasion success of this species, and cyclopoid copepods are known to be important mosquito predators^35,36^. Indeed, given that all native copepods exhibited greater interaction strengths and strong preferences towards the invasive *A. albopictus* over the native *C. pipiens*, resident predator communities may limit levels of invader success and impact. Copepods are particularly well-adapted to thrive in ephemeral aquatic habitats which these mosquitoes colonise, owing to their ability to enter dormant life history stages and spread *via* zoochorous dispersal by vectors such as birds and insects, or by wind^33,34,39,40^. Whilst human-mediated copepod introductions into minute container-style habitats have been required for effective mosquito control at community-scales^35^, even in these instances, our results provide evidence for the efficacy of copepods in reducing target invader populations whilst alleviating native species from predatory impact. Nevertheless, mechanistic interpretation of our laboratory experimental results should be cautioned in the context of invasion success, with further field-based validation required that incorporates additional context-dependencies, such as emergent effects from interactions with other predator types and habitat complexities.

In the present study, irrespective of copepod species, we consistently demonstrate higher magnitude *per capita* ecological impacts towards invasive *A. albopictus* prey as compared to analogous native *C. pipiens* prey using a comparative functional response approach. We subsequently demonstrate clear consumptive preferences towards invasive over native mosquito prey by all focal consumers regardless of proportional prey species availability, conducive with a lack of prey switching. Then, we integrate fecundity estimations as a proxy for the numerical response to quantify and compare the potential biotic resistance of resident consumers towards this invasive prey species. Whilst drawing parallels between laboratory-based studies and field observations should viewed with caution, our results align with field patterns of coexistence, wherein invasive *Aedes* mosquitoes have repeatedly been shown to coexist with native competitors, despite their clear competitive advantage^23–25^. We propose that the combination of functional and numerical responses, alongside examinations of prey switching propensities, may help to predict the occurrence of such field patterns in relation to biotic resistance and invasion success. Although we applied our metrics to a model copepod-mosquito predator-prey system, our approaches are equally applicable to other consumer-resource systems where an invasive species suffers from biotic resistance by resident consumers (e.g. predators). Thus, our metrics can, at least theoretically, be applied across multiple habitat types and taxonomic groups for predictions of invasion success and quantifications of biotic resistance. Yet, given numerous additional context-dependencies are known to alter levels of biotic resistance (e.g. habitat complexity^6,18^), these effects should be considered in future studies to better-reflect real systems.

*Macrocyclops albidus*, *M. fuscus* and *M. viridis* exhibited type II functional responses towards both prey types, characterised by high rates of mosquito prey consumption at low densities. This finding aligns with copepod functional responses forms reported in other studies^18^. However, functional response attack rates tended to be considerably higher, and handling times lower, towards invasive *A. albopictus* as compared to native *C. pipiens*. Given attack rates correspond to impacts at low prey densities whilst handling times reciprocate asymptotic maximum feeding rates, *per capita* predatory impacts towards *A. albopictus* remain higher than analogous native prey irrespective of prey density. The particularly high *per capita* impact of copepods towards *A. albopictus* also aligns with the documented ability of copepod biocontrol agents to be especially efficacious in the suppression of *Aedes* mosquitoes as compared to *Culex*^36^. Furthermore, our holistic derivations of *per capita* impact through coupling of attack rate and handling time into the functional response ratio (FRR: *a*/*h*) demonstrate greater predatory impacts by all focal copepods towards the invasive mosquito prey as compared to the native. This differential impact was particularly pronounced for *M. viridis*. The FRR metric has recently been developed for invasion scientists and practitioners and balances information from both key functional response parameters^41^. Invasive consumers have been shown to exhibit consistently higher FRRs as compared to native comparators across multiple study systems and taxonomic groups^41^. In a similar vein, we suggest that the FRR metric can be applied in quantifications of biotic resistance across study systems, as it can negate contradictory impact predictions for natural enemies based on one functional response parameter over the other (i.e. attack rate, handling time).

None of the copepod predators examined in the present study exhibited a prey switching propensity away from the invasive prey. That is, relative to proportional abundances, invasive *A. albopictus* were disproportionately selected over native *C. pipiens* across all availabilities. Prey switching facilitates patterns of coexistence in ecosystems through a form of frequency-dependent predation characterised by low density refuge effects^4,15^. If our laboratory-based results persist in the wild, it is likely that the sustained preferential selection towards *A. albopictus* would permit patterns of coexistence between these prey species even in light of the superior competitive capability of *A. albopictus* over analogous native mosquito species^24,27–29,37,42,43^. As such, this consumptive preference may offset competitive replacement of the native by the invader. Our results exemplify the potential power of our metrics for quantifications of biotic resistance which may mediate levels of invasion success, and future work should ground-truth these concepts across other invasive species study systems using field-based observations.

Behavioural responses to predator cues are likely key drivers of such differential biotic resistance between invasive and native prey. In particular, naïveté to unfamiliar predators in novel ecosystems can influence interaction strengths and further impede invasion success^20,44^. Invasive *Aedes* mosquitoes have been shown to be less responsive to predation risk and exhibit higher incidences of behaviours which make them more apparent and vulnerable to predators (e.g. thrashing, browsing)^24,25^. Indeed, whilst *Culex* mosquitoes are filter feeders which spend most time at the water surface, *Aedes* are browsers which spend more time thrashing below the surface^45^. Given these substantial behavioural differences, it is plausible that the predatory patterns exhibited by copepods in the present study extend to other aquatic predator groups, owing to potentially higher encounter rates with the more motile *Aedes* prey. Furthermore, *Aedes* mosquitoes have been shown to be attracted to predatory copepods when ovipositing, whilst *Culex* mosquitoes are evasive of these cues^18,46^. Such behavioural factors could be major drivers in limiting the success of invasive species *via* biotic resistance and, in our system, may help to regulate disease risk in the context of invasive vector mosquito species.

The present study integrated estimates of fecundity as proxies for numerical responses of copepods^47^. The use of such proxies has proven robust in derivations of ecological impacts of invasive species and biocontrol agents^3,48^, and, here, high fecundity could facilitate rapid population-level responses to increases in resource availability following natural enemy inoculation. However, importantly, the present study did not compare fecundities of predators fed on the focal invasive/native prey, which may have altered estimates given differences in nutritional values between prey species. Nonetheless, whilst *M*. *fuscus* exhibited high magnitude functional responses towards invasive prey and strong selective tendencies, the fecundity of this copepod is substantially lower than both *M. albidus* and *M. viridis*^47^. Accordingly, in this study, our novel metric identified *M. viridis* as a particularly efficacious predator towards invasive *A. albopictus* prey, owing to high *per capita* impacts towards the invader, strong selectivity traits and relatively marked fecundity. For management, our predictive metrics suggest augmentative releases of native copepods such as *M. viridis* for biocontrol could be especially efficacious in the suppression of invasive mosquito species, in light of favourable consumptive and reproductive traits.

In conclusion, we propose that the integration of traditional ecological concepts that have been neglected by invasion scientists could enhance predictions of biotic resistance and help to inform invasion success. In turn, such predictions of biotic resistance directly inform management strategies for pests, vectors and invasive species *via* biocontrol. Biotic resistance from predators can be a key mechanism which controls invasion success, and these predator-prey interactions can be robustly quantified in controlled laboratory conditions. We show that the assimilation of functional responses, numerical response proxies and prey switching propensities enables more holistic derivations of potential biotic resistance towards invasive species at the population-level. Our results are consistent with empirical patterns, whereby the invasive mosquito *A. albopictus* is capable of outcompeting native mosquito species in a laboratory setting^27,37^, but has not been able to displace native mosquitoes in a similar fashion in the field^24,49^. Yet, further field-based studies are required to validate the predation patterns documented in the present study, and link them to invasion success. Nevertheless, we propose that biotic resistance is an important factor in regulating the invasion process, and can be quantified using metrics grounded in classical ecological concepts. For practitioners, use of these concepts could enable relatively rapid comparisons of biological control agents prior to release, by quantifying and comparing *per capita* agent effects and preferences alongside population-level responses. In turn, this could improve the efficiencies associated with natural enemy introductions. Future research should also seek to ascertain the context-dependency of these approaches in predicting the success or failure of invasions across a multitude of study systems, alongside implications for the efficacy of biocontrol agents. Moreover, quantifications of predatory efficacies across a full spectrum of life history stages would provide a more holistic account of biotic resistance levels.

## Materials and Methods

### Animal collection and husbandry

Eggs of *Aedes albopictus* were obtained through the infraVec2 project and originated from Montpellier, France. Eggs of *Culex pipiens* complex mosquitoes were obtained from a colony maintained at Queen’s Marine Laboratory (QML), Portaferry, Northern Ireland, originating from The Pirbright Institute, Surrey, England^48^. Both mosquito species were maintained in a laboratory at QML, at 25 ± 2 °C and under a 16:8 light and dark photoperiod. The focal predators, *Macrocyclops albidus*, *Macrocyclops fuscus* and *Megacyclops viridis* were obtained from Glastry Clay Pit Ponds, Northern Ireland (54°29′18.5″N, 5°28′19.9″W) and cultured in the same laboratory (25 ± 2 °C,16:8 light and dark) on a diet of *Paramecium caudatum* and *Chilomonas paramecium ad libitum* until maturation.

### Experimental design

Adult female predatory *M. albidus*, *M. fuscus* and *M. viridis* (respective mean total lengths excluding caudal setae ± SD: 1.70 ± 0.09 mm; 1.81 ± 0.11 mm; 1.83 ± 0.17 mm) were selected for experiments and separately starved for 24 h prior to feeding. Recently hatched, size-matched first instar *A. albopictus* (mean ± SD: 1.40 ± 0.12 mm) and *C. pipiens* (mean ± SD: 1.32 ± 0.11 mm) larvae were used as prey. Copepods are known to be most efficient in consumption of early instar mosquito prey^36^. Experiments were undertaken in 20 mL arenas of 42 mm dia. containing dechlorinated tapwater from an aerated source during daylight. We employed a phenomenological experimental approach to compare biotic resistance towards mosquitoes factorially in a replicated laboratory design. Accordingly, our design does not seek to mechanistically replicate natural systems (see^9^). Indeed, mechanistic interpretation of such experiments must be approached with caution, or supported with further empirical parameter estimates^50–52^. Nevertheless, phenomenological designs, such as ours, are useful for comparative purposes in factorial experiments to examine differences in predator-prey interactions under controlled conditions^53^.

For the functional response experiment, prey species were introduced separately at each of five densities into arenas (2, 4, 7, 10 or 15; *n* = 5 per experimental group). For the prey switching experiment, prey species were introduced in combination at each of five ratios (2:18, 5:15, 10:10, 15:5 or 18:2; *n* = 3 per experimental group). Experiments were conducted in a completely randomised array to eliminate positional effects. After addition, prey were allowed to settle for 2 h prior to the beginning of the experiments *via* predator introduction. Once individual copepod predators were introduced, they were allowed to feed for 6 h, after which the predators were removed and remaining live larval mosquito prey counted and identified to quantify numbers killed. Controls in each experiment consisted of a replicate of each prey treatment in the absence of predators.

### Statistical analyses

Data were analysed using R v 3.5.1^54^. In the functional response experiment, raw numbers of prey consumed were examined with respect to prey species, predator species and starting prey densities in a factorial generalised linear model (GLM). All interaction terms were included in the initial model. A Poisson error distribution with log link was employed. We used second-order derivations of Akaike’s Information Criterion (AICc) and model averaging to identify the best-supported model using the ‘MuMIn’ package^55,56^. Here, all possible models were identified and ranked based on AICc (lower values indicate a better fit). Model comparisons used ∆AICc, comprising the difference between the AICc of candidate models and the best-supported model. Akaike model weights (*w*_*i*_) were additionally used to probabilistically identify the best model, wherein predictor variables with good support yielded high cumulative *w*_*i*_ values (near 1). *Post-hoc* Tukey tests were performed using ‘lsmeans’ where a factor yielded significance at the 95% confidence interval^57^.

The ‘frair’ package was used to perform functional response analyses^58^. Logistic regression considering the proportion of prey consumed with respect to initial prey density was used to identify functional response types. Categorically, a type II functional response is inferred where a significantly negative first-order term results, whilst a significantly positive first order term followed by a significantly negative second-order term indicates a type III functional response^59^. To account for prey depletion over the experimental period, we fit Rogers’ random predator equation for the non-replacement of prey^59,60^:

1 $${N}_{e}={N}_{0}(1-\exp (a({N}_{e}h-T)))$$

where *N*_*e*_ is the number of prey eaten, *N*_0_ is the initial density of prey, *a* is the attack rate, *h* is the handling time and *T* is the total experimental period. The random predator equation was fit for each predator and prey treatment group using maximum likelihood estimation, with the *Lambert W* function implemented to make the equation solvable^61^. Functional response models were non-parametrically bootstrapped 2000 times to generate 95% confidence intervals around starting estimations. Using the handling time (*h*) parameter, maximum feeding rate estimates (1/*h*) were additionally calculated.

We subsequently applied a new overall measure of *per capita* impact towards both prey types for each predator, by combining attack rates (*a*, functional response initial slope) and handling times (*h*, functional response asymptote) into the functional response ratio, which captures both parameters^41^:

2 $${\rm{FRR}}=a/h$$

where FRR is the attack rate *a* divided by the handling time *h*. This solves the problem of which parameter to choose for comparisons, as a large *a* combined with a small *h* gives a large value (and hence quantifies a large *per capita* effect), while a low *a* and a high *h* gives a low value (and hence quantifies a low *per capita* effect). We denote FRR_*i*_ as towards invasive *A. albopictus* and FRR_*n*_ as towards native *C. pipiens*.

In the prey switching experiment, numbers of prey consumed were analysed using generalised linear mixed models (GLMM) with Poisson error distribution and log link using the ‘lme4’ package^62^. Here, consumption was modelled with prey species, predator species and proportion available, alongside their interactions, as fixed effects, and with a random effects structure to account for repeated measures of prey types within each experimental replicate. Model averaging based on AICc was, again, implemented to select predictors which minimised information loss^55^, and *post-hoc* comparisons were performed using pairwise Tukey tests^57^.

Manly’s selectivity index was then used to quantify preferences for invasive *A. albopictus* prey by each predator species across proportions available, with adjustments for non-replacement of prey^63,64^:

3 $${\alpha }_{i}=(\mathrm{ln}(({n}_{i0}-{r}_{i})/{n}_{i0}))/{\sum }_{j=1}^{m}(\mathrm{ln}(({n}_{n0}-{r}_{n})/{n}_{n0}))$$

where *a*_*i*_ is Manly’s selectivity index for invasive *A. albopictus*, *n*_*i*0_ is the number of the invader available at the start of the experiment, *r*_*i*_ is the number of the invader consumed, *m* the number of prey types, *n*_*n*0_ the number of native *C. pipiens* available at the start of the experiment and *r*_*n*_ is the number of native prey consumed. Resulting indices range from 0 to 1, wherein 0 indicates complete avoidance and 1 indicates complete preference. In our two-prey system, values of 0.5 are indicative of neutral selectivity by predators between prey types. Prior to formal analysis, we transformed resulting *a*_*i*_ values to account for extreme data points (0, 1)^65^:

4 $${\alpha }_{t}=({\alpha }_{i}(n-1)+0.5)/n$$

where *α*_*t*_ is the transformation and *n* is the sample size. Beta regression using the ‘betareg’ package was used to compare indices towards *A. albopictus* with those expected under null preference (0.5) with respect to predator species and prey proportion available^66^. Model averaging based on AICc was used in model selection as before, and *post-hoc* comparisons were undertaken using Tukey tests^55,57^.

Combining the above results, we then quantified Biotic Resistance (BR) towards invasive prey using relative FRRs between invasive *A. albopictus* and native *C. pipiens* prey (FRR_*i*_/FRR_*n*_; Eq. 2), reproductive effort as a numerical response proxy (clutch weight produced per female body weight per day^47^) and mean invasive prey preferences (Eq. 3) for each predator species:

5 $${\rm{BR}}=({{\rm{FRR}}}_{i}/{{\rm{FRR}}}_{n})\times {\rm{FE}}\times {a}_{i}$$

where the Biotic Resistance (BR) of a predator towards invasive prey is a product of the relative FRR between invasive and native prey (FRR_*i*_/FRR_*n*_), predator numerical response proxy reproductive effort (fecundity, FE) and the mean preference index towards the invasive prey (*α*_*i*_). We selected fecundity as a suitable numerical response proxy given its importance for the proliferation of natural enemies following changes in resource availability, and because reproductive effort estimates for the focal predator species were readily available in the literature. Relative Biotic Resistance (RBR) was then developed and used to compare among the three different predator species:

6 $${\rm{RBR}}={\rm{BR}}1/{\rm{BR}}2$$

where BR1 and BR2 are Biotic Resistance for predator 1 and predator 2, respectively. Here, values of 1 indicate equivalence in biotic resistance between the two predators and values >1 indicate greater biotic resistance by predator 1 as compared to predator 2. Conversely, RBR values <1 indicate lesser biotic resistance by predator 1 compared to predator 2. We produced triplots to further illustrate differences^16^.

We thus first quantified and compared functional responses by three native predators towards native and invasive prey when presented separately. Second, we examined prey preferences of the same predator species towards the two prey species when both are present simultaneously at different relative proportions. Thirdly, we used a predator numerical response proxy (fecundity), alongside functional responses and prey preferences, to predict which resident predator is likely to exert the greatest degree of biotic resistance towards the focal invasive species.

## Supplementary information

Supplementary Table 1

Dataset 1

Dataset 2

## Data Availability

Underlying functional response and prey switching data are available in the online supporting information.
