# Supplementary material for: A novel metric reveals biotic resistance potential and informs predictions of invasion success
Source: Sci Rep. 2019 Oct 25;9:15314. doi: 10.1038/s41598-019-51705-9 (PMC6814831; doi:10.1038/s41598-019-51705-9)
Supplement: Supplementary file 1 — Supplementary Table 1 [file 41598_2019_51705_MOESM1_ESM.docx]

**A novel metric reveals biotic resistance potential and informs predictions of invasion success**

Ross N. Cuthbert*^1, 2^, Amanda Callaghan^2^, Jaimie T. A. Dick^1^

^1^*Institute for Global Food Security, School of Biological Sciences, Queen’s University Belfast, Belfast BT9 5DL, UK*

^2^*Ecology and Evolutionary Biology, School of Biological Sciences, University of Reading, Harborne Building, Reading RG6 6AS, UK*

****Corresponding author email****: rcuthbert03@qub.ac.uk*

Table S1. Model averaging results of generalised linear model (GLM, a), generalised linear mixed model (GLMM, b) and beta regression (betareg, c) on: (a) raw consumption from functional response experiment as a function of prey species (2 levels), predator species (3 levels) and prey density (5 levels); (b) raw consumption from prey switching experiment as a function of prey species (2 levels), predator species (3 levels) and prey proportion (5 levels), and; (c) prey preference indices as a function of index type (2 levels), predator species (3 levels) and prey proportion (5 levels). ∆AICc is the difference between the focal model and the model with the lowest AICc, weight *w_i_* is the probability that the focal model is the top model and cum. *w_i_* demarks cumulative model weights. Models with high importance (∆AICc < 2) are shown here.

| Response | Model | df | logLik | AICc | ∆AICc | *w_i_* | Cum. *w_i_* |
| --- | --- | --- | --- | --- | --- | --- | --- |
| (a) Functional response consumption (GLM) | Prey + Predator + Density | 8 | -255.37 | 527.76 | 0.00 | 0.53 | 0.53 |
|  | Prey × Predator + Density | 10 | -253.27 | 528.13 | 0.37 | 0.44 | 0.97 |
| (b) Prey switching consumption (GLMM) | Prey + Predator + Proportion | 11 | -145.49 | 316.36 | 0.00 | 0.58 | 0.58 |
|  | Prey + Proportion | 9 | -148.90 | 318.06 | 1.70 | 0.25 | 0.83 |
| (c) Preference indices (betareg) | Index × Proportion | 11 | 27.01 | -28.64 | 0.00 | 0.88 | 0.88 |
